# Investigating the role of super-enhancer RNAs underlying embryonic stem cell differentiation

**DOI:** 10.1186/s12864-019-6293-x

**Published:** 2019-12-30

**Authors:** Hao-Chun Chang, Hsuan-Cheng Huang, Hsueh-Fen Juan, Chia-Lang Hsu

**Affiliations:** 1 Graduate Institute of Biomedical Electronics and Bioinformatics, National Taiwan University, Taipei, Taiwan; 2 Institute of Biomedical Informatics, National Yang-Ming University, Taipei, Taiwan; 3 Department of Life Science, National Taiwan University, Taipei, Taiwan; 4 Department of Medical Research, National Taiwan University Hospital, Taipei, Taiwan; 5 Graduate Institute of Oncology, National Taiwan University College of Medicine, Taipei, Taiwan

**Keywords:** Enhancer RNA, Super-enhancer, Embryonic stem cell, Cell differentiation

## Abstract

**Background:**

Super-enhancer RNAs (seRNAs) are a kind of noncoding RNA transcribed from super-enhancer regions. The regulation mechanism and functional role of seRNAs are still unclear. Although super-enhancers play a critical role in the core transcriptional regulatory circuity of embryonic stem cell (ESC) differentiation, whether seRNAs have similar properties should be further investigated.

**Results:**

We analyzed cap analysis gene expression sequencing (CAGE-seq) datasets collected during the differentiation of embryonic stem cells (ESCs) to cardiomyocytes to identify the seRNAs. A non-negative matrix factorization algorithm was applied to decompose the seRNA profiles and reveal two hidden stages during the ESC differentiation. We further identified 95 and 78 seRNAs associated with early- and late-stage ESC differentiation, respectively. We found that the binding sites of master regulators of ESC differentiation, including NANOG, FOXA2, and MYC, were significantly observed in the loci of the stage-specific seRNAs. Based on the investigation of genes coexpressed with seRNA, these stage-specific seRNAs might be involved in cardiac-related functions such as myofibril assembly and heart development and act in *trans* to regulate the co-expressed genes.

**Conclusions:**

In this study, we used a computational approach to demonstrate the possible role of seRNAs during ESC differentiation.

## Background

During embryonic development and cellular differentiation, distinct sets of genes are selectively expressed in cells to give rise to specific tissues or organs. One of the mechanisms controlling such highly organized molecular events are enhancer–promoter contacts [1]. The disruption of enhancer–promoter contacts can underlie disease susceptibility, developmental malformation, and cancers [1, 2]. In addition, a cluster of enhancers speculated to act as switches to determine cell identity and fate is named the ‘super-enhancer’ [3–5]. Super-enhancer is generally characterized as a class of regulatory regions that are in close proximity to each other and densely occupied by mediators, lineage-specific or master transcription factors, and markers of open chromatin such as H3K4me1 and H3K27ac [3]. Under the current definition, super-enhancers tend to span large genome regions, and several studies have reported that they tend to be found near genes that are important for pluripotency, such as OCT4, SOX2, and NANOG [6, 7].

Recently, a class of noncoding RNAs transcribed from the active enhancer regions has been recognized due to advances in sequencing technology, and termed enhancer RNAs (eRNAs). Because enhancers tend to be tissue- and state-specific, eRNAs derived from the same enhancers may differ across tissues [8], and the same stimulation could induce the production of eRNAs via divergent signaling pathways [9]. Although the functions and regulation mechanisms of these eRNAs are unclear, they may play an active role in the transcription of nearby genes, potentially by facilitating enhancer–promoter interactions [10], and the abnormal expression of eRNAs is associated with various human diseases [11].

Although several studies have shown that eRNAs are associated with super-enhancer regions [12–14], no work has yet been done to investigate the role of super-enhancer RNAs (seRNAs) during embryonic stem cell differentiation. Here, we propose a computational approach to characterize seRNAs based on eRNA profiles derived from cap analysis gene expression sequencing (CAGE-seq) and identify stage-specific seRNAs using non-negative matrix factorization (NMF). A previous study has used NMF to dissect seRNA profiles and found that different cell types were well classified, suggesting seRNA expression is associated with the determination of cell fate [15]. In this study, we ask if seRNAs play a critical role during the embryonic stem cell (ESC) differentiation. We analyzed the seRNA profiles by NMF to determine the hidden stages during ESC differentiation. Finally, we identified the stage-specific seRNAs and further investigated their functional roles via their co-expressed genes.

## Results

### Identification of super-enhancer RNAs underlying the differentiation of embryonic stem cells

To investigate seRNAs during embryonic differentiation, we used time-resolved expression profiles of embryonic stem cells (ESCs) from the FANTOM5 project, which were profiled using CAGE-seq techniques [16]. These datasets contain 13 time-points (range: 0–12 days) and provide expression profiles for both mRNAs and eRNAs during differentiation from ESCs to cardiomyocytes. After removal of lowly expressed eRNAs, there were 28,681 expressed eRNAs during differentiation from ESCs to cardiomyocytes qualified and quantified by CAGE-seq.

The typical approach for super-enhancer identification is to stitch together enhancer regions within 12.5 kb of each other and analyze the ChIP-seq binding patterns of active enhancer markers using the Rank Ordering of Super-enhancers (ROSE) algorithm [6]. However, it is unclear whether seRNAs inherit these properties. To address this issue, we used the expression values of unstitched and stitched eRNAs and identified seRNAs by ROSE algorithm. We combined the eRNAs that located within 12.5 kb of each other into a single larger eRNA [6], and obtained 16,990 stitched eRNAs containing median of 1 expressed eRNA (range: 1–155).

**Fig. 1:**
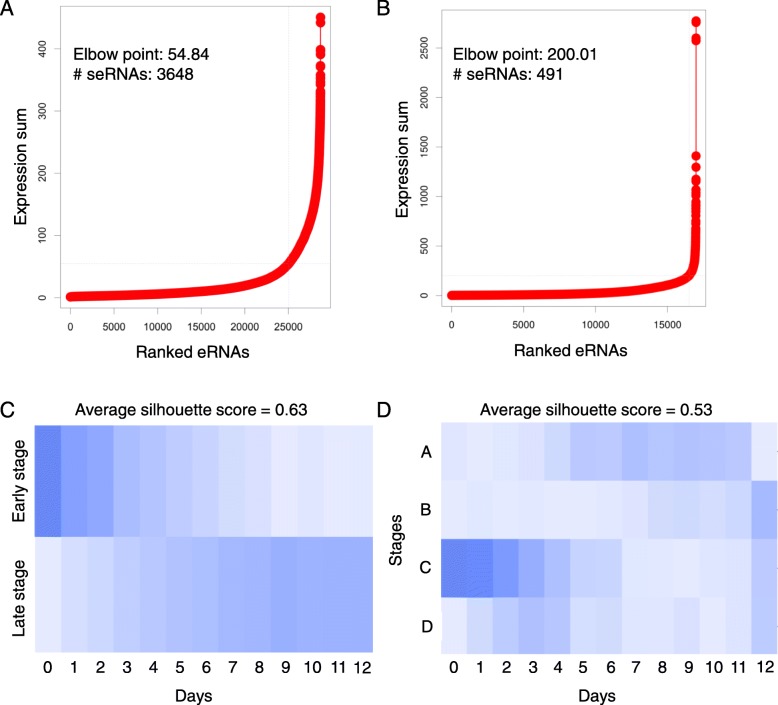
Super-enhancer RNA identification and NMF decomposition of time-coursed ESC differentiation to cardiomyocytes. **a** and **b** Ranking of unstitched (left) and stitched enhancers (right) based on the expression values. **c** and **d** Stage to sample matrix of the decomposition from the unstitched (left) and stitched super-enhancer RNA profiles (right)

To determine the seRNAs, we performed the ROSE algorithm on unstitched and stitched eRNAs, respectively. Briefly, the unstitched and stitched eRNAs were each ranked on the basis of corresponding expression values, and their expression values were plotted (Fig. 1a, b). These plots revealed a clear point in the distribution of eRNAs where the expression value began increasing rapidly, and this point was determined by a line with a slope of one was tangent to the curve. eRNAs that were plotted to the right of this point were designated as seRNAs. Altogether, 3648 and 491 (median of 4 expressed eRNAs, range: 1–155) seRNAs were identified from the unstitched and stitched enhancer regions, respectively.

To identify stage-specific seRNAs, first, the non-negative matrix factorization (NMF) was employed to decompose the seRNA expression profiles and identify hidden stages during the differentiation of ESCs to cardiomyocytes. We performed the NMF with different number of stages (from 2 to 12), and evaluated the clustering performance by computing silhouette scores (good cluster have higher silhouette scores). On the basis of the best average silhouette scores (Additional file 1: Figure S1), two and four stages were determined for unstitched and stitched seRNA expression profiles, respectively. We can assign each time point into a stage based on the values in the stage vs. sample matrix decomposed from NMF (Fig. 1c,d). We noted that the expression profile of the unstitched enhancers achieved a higher average silhouette score than that of the stitched enhancers. In addition, the stages determined from the unstitched enhancers appear to delineate the boundary between the day 0–4 (named early stage) and day 5–12 (named late stage) of differentiation (Fig. 1c). Although there were four stages determined from the stitched seRNA profiles, the samples could majorly be classified into early- (Stage C: day 0–4) and late-stage (Stage A: day 5–11 and Stage B: day 12), consistent with the result of unstitched seRNAs. Therefore, we focused on the seRNAs derived from unstitched enhancer regions. Next, according to the result of NMF, the stage-specific seRNAs were determined by comparing the expression values between two stages. Finally, there were 95 and 78 seRNAs active in the early and late stages of ESC differentiation, respectively (Additional file 2).

### Transcription factors driving expression of stage-specific seRNAs

**Fig. 2:**
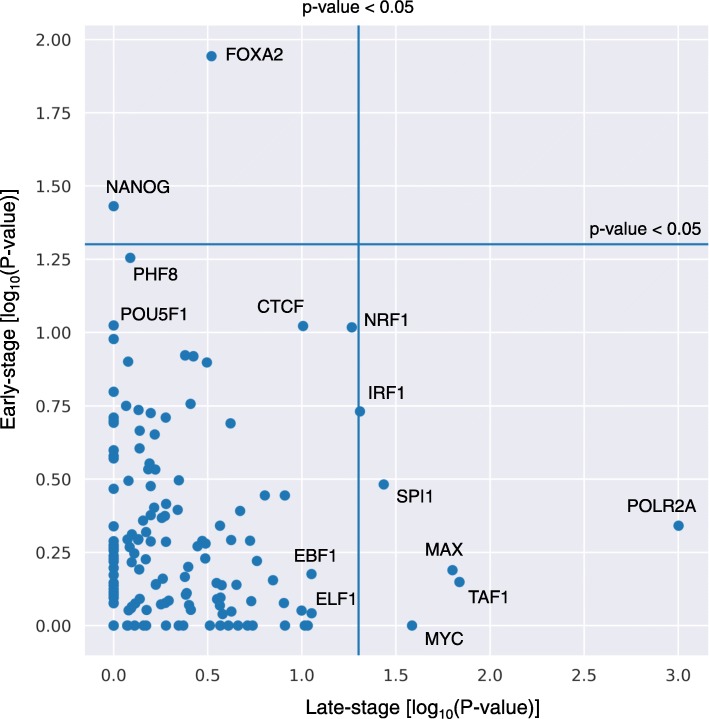
Enrichment of transcription factors associated with stage-specific super-enhancer RNAs. Scatter plot showing the over-representation analysis *P*-values for each TF. Significantly enriched TFs and some nearly significant TFs are annotated with their gene symbols

A primary role of transcription factors (TFs) is the control of gene expression necessary for the maintenance of cellular homeostasis and the promotion of cellular differentiation. To investigate the association between stage-specific seRNAs and TFs, TF over-representation analysis was performed to assess whether these seRNA loci are unexpectedly bound by TFs (Fig. 2). In early stage of ESC differentiation, stage-specific seRNAs were significantly driven by NANOG and FOXA2. Indeed, NANOG is a master TF of ESC pluripotency [17]. Additionally, although FOXA2 is not a master TF of ESC differentiation, it is strongly upregulated during the early stages of endothelial differentiation [18]. In contrast, besides MYC/MAX complexes, more basal TFs involved in the maintenance of cellular states were enriched in the late-stage seRNAs: POLR2A, TAF1, SPI1, and IRF1.

### Inference of seRNA functions from the seRNA-associated genes

**Fig. 3:**
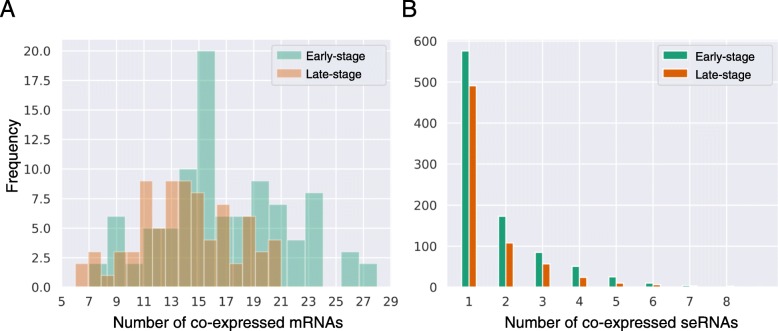
Distribution of interactions in the seRNA–mRNA co-expression network. **a** The distribution of the numbers of co-expressed mRNAs above the cutoff. **b** The distribution of the number of co-expressed seRNAs

Although the functional roles of eRNAs remain unknown, we can investigate the possible role of seRNAs using their co-expressed mRNAs [19, 20]. We hypothesized that the co-expressed genes imply the possible mechanisms of seRNA-mediated regulation and tend be involved in similar biological pathways or processes. We performed a co-expression analysis of seRNAs and mRNAs to determine the seRNA-associated genes. To determine the seRNA-coexpressed mRNAs, the Pearson’s correlation coefficient among seRNAs and mRNAS were calculated and then converted into the mutual rank [21]. A mRNA with mutual ranks to seRNAs of ≤5 was considered as a seRNA-associated mRNA. Each seRNA was found to have a median of 15 associated mRNAs (range: 6–28), but most of the mRNAs were co-expressed with a seRNA, suggesting that a given set of genes is regulated by a specific enhancer–promoter loop (Fig. 3a,b).

**Fig. 4:**
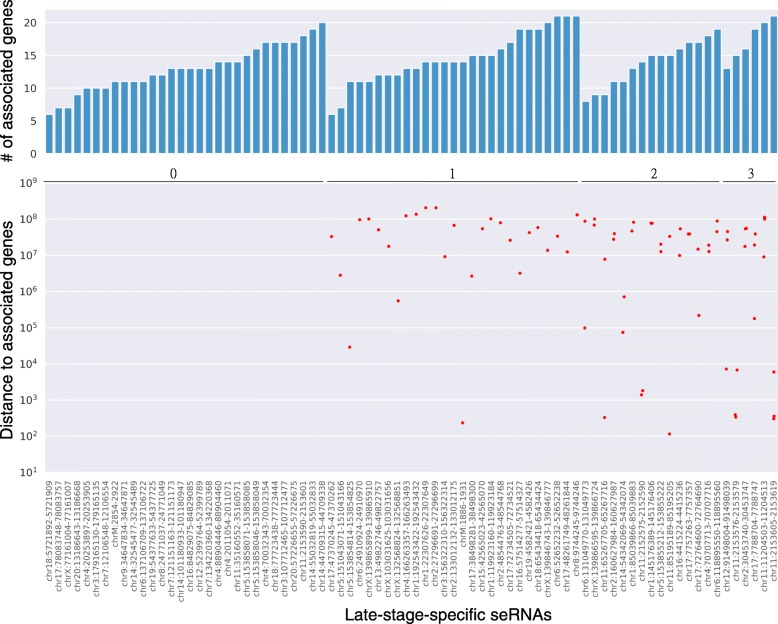
Location distribution of associated genes for late-stage-specific seRNAs. Bar plot showing the number of associated genes and scatter plot showing the distance between associated genes and their seRNAs. The distance is defined as the absolute difference between two locus midpoints. The number of associated genes located on the same chromosome as their seRNA is indicated above the scatter plot

Even though a few cases in which the enhancers act in *trans* were observed [22], most of them act in *cis* (i.e., the enhancers and their cognate genes are located on the same chromosome). In addition, several studies show that the level of expression of eRNAs is positively correlated with the expression level of genes near their corresponding enhancer [10, 23, 24]. However, we examined the genomic distance between seRNAs and their corresponding associated genes and found that most seRNA–mRNA pairs are not located on the same chromosome (Fig. 4 and Additional file 1: Figure S2). In addition, even though other seRNA–mRNA pairs are on the same chromosome, the genomic distances between them are up to 10,000 kb (Fig. 4 and Additional file 1: Figure S2). This suggests the possibility that seRNAs might act in *trans* or trigger pathway activity, leading to the expression of distal genes.

**Fig. 5:**
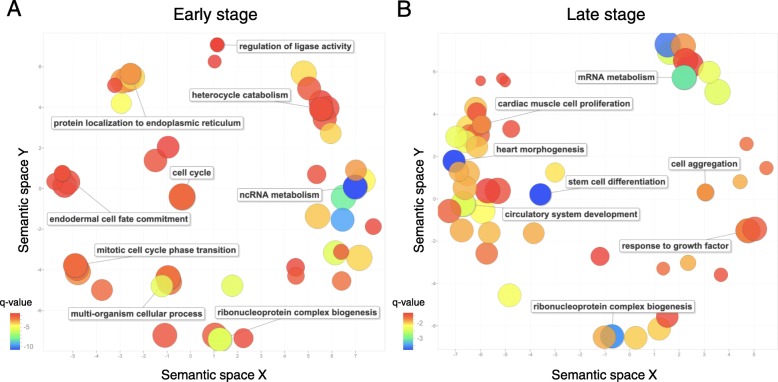
The statistically over-represented GO terms within genes related to early- and late-stage-specific seRNAs. The scatter plots generated by REVIGO show the cluster representatives in a two dimensional space derived by applying multidimensional scaling to a semantic similarity matrix of GO terms for early- (**a**) and late-stage-specific seRNAs (**b**). Bubble color indicates the q-value of GO over-representation analysis and size indicates the frequency of GO term used in human genome. Names of several cluster representatives are shown

To examine the global functions of stage-specific seRNAs, Gene Ontology (GO) over-representation analysis using topGO [25] was applied to the genes associated with early- or late-stage-specific seRNAs, respectively. The GO terms with q-value < 0.05 were visualized as a scatter plot via REVIGO. Interestingly, the genes associated with early-stage-specific seRNAs are related to the process of cell proliferation (such as cell cycle, q-value = 0.004) and determination of cell fate (such as endodermal cell fate commitment, q-value = 0.016) (Fig. 5a and Additional file 3), whereas late-active seRNAs are associated with genes involved in stem cell differentiation (q-value = 0.0002) and heart morphogenesis (q-value = 0.0002) (Fig. 5b and Additional file 4).

### Stage-specific seRNAs bound by TFs are associated with important cardiac genes

**Fig. 6:**
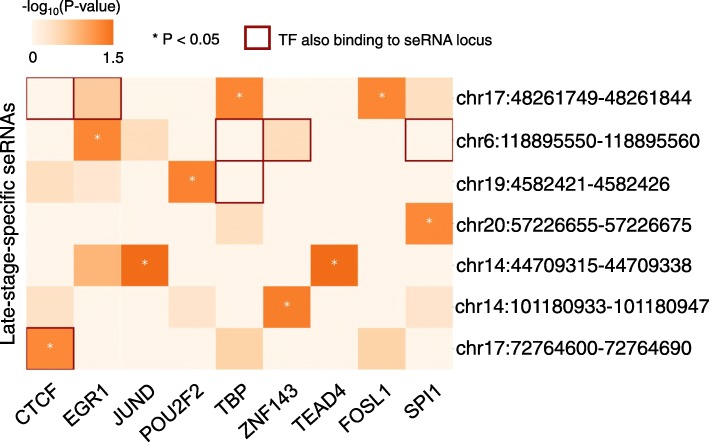
The regulator binding matrix of late-stage-specific seRNA-associated genes. Heatmap visualizing the results of TF over-representation analysis on seRNA-associated genes. Red borders indicate that the TF also binds to the super-enhancer. The color denotes −log_10_ of the P-value obtained by the Fisher’s exact test. (* *P* < 0.05)

**Fig. 7:**
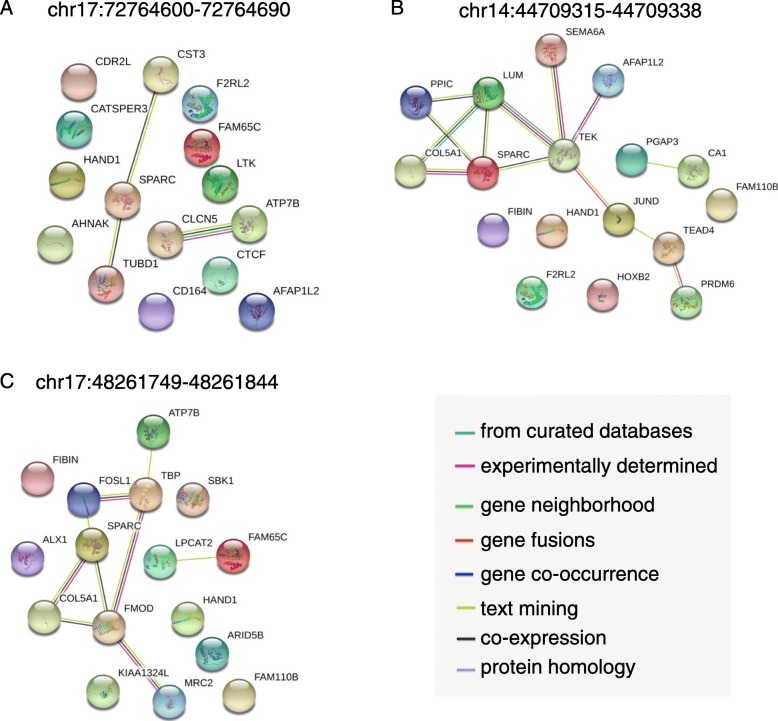
The protein–protein interaction (PPI) network of late-stage-specific seRNA-associated genes. The PPI network obtained from STRING ver.10.5, in which each node is a protein and each edge has a different kind of evidence of interaction. Captions are the loci of super-enhancers. Driving TFs were also included in the network: **a** chr17:72764600–72,764,690: CTCF; **b** chr14:44709315–44,709,338: JUND and TEAD4; and **c** chr17:48261749–48,261,844: FOSL1 and TBP

Next, we examined seRNAs individually by performing TF and GO over-representation analyses on each set of seRNA-associated genes. We found that each of these sets was mediated by different regulators, and in some cases, the regulator mediated not only its associated genes but also the seRNA itself (Fig. 6 and Additional file 1: Figure S3). For example, a late-stage-specific seRNA (chr17:72764600–72,764,690) located in close proximity to solute carrier family 9 member 3 regulator 1 (SLC9A3R1) has a CTCF binding site within its locus and the promoters of its associated genes show enrichment for CTCF (Fig. 6). We further examined the CTCF ChIP-seq performed on human ESCs and the derived cells [26], and found a stronger CTCF binding signal on this seRNA locus in ESCs, compared to other ESC-derived cells (Additional file 1: Figure S4). The functions of these seRNA-associated genes are related to embryonic heart tube formation and ion transmembrane transport (Fig. 7 and Additional file 5). Indeed, CTCF is required during preimplantation embryonic development [27], and several ion transporter genes, such as CLCN5 and ATP7B, are expressed to maintain the rhythmicity and contractility of cardiomyocytes [28].

Besides the seRNA located at chr17:72764600–72,764,690, we did not find any TFs that both bind to late-stage seRNA loci and are enriched for the promoters of the corresponding associated genes (Fig. 6). However, two seRNAs might be important for ESC differentiation. For the seRNA at chr14:44709315–44,709,338, JUND and TEAD4 binding sites were unexpectedly observed in the promoters of its associated genes (both *p*-values < 0.05, Fisher’s exact test). JUND is a critical TF in the limiting of cardiomyocyte hypertrophy in the heart [29], whereas TEAD4 is a muscle-specific gene [30]. There were strong functional associations among these associated genes (Fig. 7b) and the functions of these associated genes are significantly related to cardiovascular system development and the organization of collagen fibrils (Additional file 5). In the developing cardiovascular system, LUM (lumican) and COL5A1 (collagen type V, alpha 1) can participate in the formation of collagen trimers, which are required for the elasticity of the heart septa [31]. In addition, SPARC exhibits calcium-dependent protein–protein interaction with COL5A1 [32]. The other seRNA, which is located at chr17:48261749–48,261,844 near the type-1 collagen gene (COL1A1), has two enriched TFs: FOSL1 and TBP (Fig. 6). FOSL1 is a critical regulator of cell proliferation and the vasculogenic process [33] and is a component of the transcriptional complex AP-1, which controls cellular processes related to cell proliferation and differentiation [34]. TBP is a general TF that helps form the RNA polymerase II pre-initiation complex. The interactions among these associated genes show that FMOD may cooperate with TBP to promote the differentiation of mesenchymal cells into cardiomyocytes in the late stages of cardiac valve development [35] (Fig. 7c). This group of seRNA-associated genes also includes SPARC and COL5A1, suggesting a similar role to the seRNA located within chr14 mentioned above. These two cases reveal that these seRNAs might be involved in cardiomyocyte differentiation, but whether seRNAs play as a key regulator have to be further experimentally validated.

Although we did not find any super-enhancer–promoter loops driven by TFs, we identified one group driven by a key regulator that has functions critical for cardiomyocytes. We also found two groups of seRNA-associated genes, which include many genes critical for cardiomyocyte formation and are driven by multiple TFs. Despite the connection between late-stage-specific seRNAs and cardiomyocyte differentiation, the early-stage-specific seRNAs do not have any obvious association with cardiac-related functions (Additional file 1: Figure S3 and Additional file 6). The possible reason is that the early stage corresponds to the time before commitment during human ESC differentiation into cardiac mesoderm (about day 4) [36]. Therefore, the cells may not express cardiac-related genes during that period.

## Discussion

Super-enhancers, which are defined by a high occupancy of master regulators, have been studied by many researchers in order to exploit their functions and regulatory mechanisms. However, these studies did not take enhancer RNAs (eRNAs) into account. Therefore, we employed a novel approach and defined super-enhancer RNAs (seRNAs) based on their RNA expression levels. To justify the identification of hidden stages of ESC differentiation and the selection of stage-specific seRNAs, we demonstrated that our selected stage-specific seRNAs are significantly bound by key transcription factors and related the result to the possible roles of each differentiation stage.

The definition of super-enhancer is still ambiguous [3]. In general, the term ‘super-enhancer’ refers to an enhancer cluster with high density of active markers. Actually, a few identified super-enhancers contain single enhancers [6]. Therefore, the impact of super-enhancer on gene regulation might be its activity, not size. In this study, we identified seRNAs from stitched and unstitched eRNAs based on the procedure of the ROSE algorithm and determine the differentiation stages by the decomposition of NMF on unstitched and stitched seRNA profiles. Although there is a slight difference between the results of the unstitched and stitched seRNAs, the major two stages of ESC differentiation could be identified by both datasets (Fig. 1c and d). However, it seems that unstitched seRNAs have better discriminatory ability, compared to the stitched seRNAs. The possible reasons include each eRNA may have independent functional role [37] and some eRNAs may act *in trans*, different from enhancers [11]. The definition of seRNAs used in this work differs from the general definition of super-enhancer, but the further function and regulatory analyses of these identified seRNAs reveal these seRNAs have the similar capacity of super-enhancers during ESC differentiation [38, 39].

To infer the functions of stage-specific seRNAs, we investigated the associations between them and their co-expressed mRNAs. We found that the co-expressed mRNAs had annotated functions related to the formation of cardiomyocytes. Some key regulators bind to both super-enhancers and their associated genes, and the encoded proteins form a significant interaction network. These results suggest that the stage-specific seRNAs contribute to ESC differentiation. However, the analysis was only performed on ESC differentiation profiles and correlations among genes and thus does not reveal true interactions. More evidence is required to conclusively report the functions of seRNAs.

Genomic distances between the loci of seRNA–mRNA co-expression pairs raised a question about the possible *trans*-acting property of seRNA. In an attempt to assess whether seRNA exhibits *trans*-acting regulation activity towards its target genes, we analyzed the seRNAs with functional homogeneity and co-regulation based on their associated genes. Although we cannot prove the *trans*-acting property of seRNA, we nevertheless propose this as a potential avenue for future research.

However, the functions and regulatory mechanisms of seRNA remain obscure, and more evidence is needed due to the complexity of gene regulation. Since seRNAs are expressed in a cell-specific manner [6], and cells regulate their gene expression in many implicit ways, we propose the computational approach employed in this study to help others explore the intricate nature of seRNAs. In the meantime, various other approaches can also be adopted, such as modeling hidden stages using a nonlinear method known as auto-encoder, in addition to other methods for the construction of co-expression network to identify more informative associations.

## Conclusions

Using a computational approach, we identified and demonstrated the importance of stage-specific seRNAs. One stage-specific seRNA is driven by the same TF as its associated genes, and two seRNAs are driven by multiple TFs. All of these seRNAs are significantly bound by TFs related to cardiac muscle development. The associated genes also perform critical functions in heart development. Based on the genomic distance between co-expression pairs, we propose the possibility that seRNA might act in *trans* during regulation. Although our analysis cannot conclusively verify this property, we have provided an exploratory resource and approach for further investigation.

## Methods

### Expression data preprocessing

The time-resolved expression profiles of ESCs during the process of differentiation into cardiomyocytes were downloaded from FANTOM5. Genes and eRNAs with counts of zero in more than 75% of samples were discarded. The expression values were transformed by log_2_ and normalized using the upper-quartile normalization method. Finally, the expression levels were averaged across replicates.

### Stitching enhancer regions

Enhancer regions on the same chromosome were stitched together if they were within 12.5 kb of each other. In the case of genes located within the stitched regions, these enhancer regions were kept separate. We constructed a graph in which nodes denote enhancer regions and edges connect enhancers located within 12.5 kb, and identified the connected components of the graph. The enhancer regions within connected components of the graph were stitched together. The expression levels of the stitched enhancers were determined as the sum of the expression levels of the individual enhancers.

### Identification of seRNAs

We used the Rank Ordering of Super-enhancers (ROSE) algorithm [3] to identify active seRNAs. Briefly, the eRNAs from the unstitched or stitched enhancer regions were ranked by their expression level, and in the plots the *x*-axis is the rank of the eRNAs and the *y*-axis is the corresponding expression level. To determine this ‘elbow point’, the data were scaled such that the x and y axis were from 0 to 1 and the point for which a line with a slope of 1 was tangent to the curve was found. eRNAs above this point were defined as super-enhancer RNAs (seRNAs) and eRNAs below that point were typical eRNAs.

### Identification of differentiation stages using NMF

We derived the differentiation stages by applying NMF to the seRNA expression profiles. NMF is a dimension-reduction technique and can identify hidden stages in data by specifying the number of stages (*k*). We factorized the seRNA expression profiles (*V*) into two matrices, *W* (stage vs. sample matrix, *m x k*) and *H* (seRNA vs. stage matrix, *k x n*), such that:

$$ V\approx WH $$

Here, we determine the *W* and *H* matrices by minimizing the cost function [40]:

$$ f\left(W,H\right)\equiv \frac{1}{2}\parallel V- WH{\parallel}^2,\kern0.5em {W}_{ia}\ge 0,{H}_{bj}\ge 0,\forall i,a,b,j $$

We performed the NMF using the function implemented by the python package scikit-learn with following parameters: init = ‘nndsvd’, tol = 0.0001, max_iter = 200, alpha = 0.0, l1_ration = 0.0, and shuffle = False. Since the number of hidden stages (*k*) is a hyperparameter, we used the average of the silhouette scores to find the optimized number of hidden stages. The definition of the silhouette score for each sample is as follows [41]:

$$ \frac{b-a}{\mathit{\max}\left(a,b\right)} $$

where *a* is the mean of the intra-cluster distance and *b* is the mean of the nearest-cluster distance. The distance used here is the Euclidean distance between sample based on stage vs. sample matrix. The silhouette score ranges from − 1 to + 1, and a high silhouette score indicates that the sample is well matched to its own cluster and poorly matched to neighboring clusters. We calculated the average silhouette score for *k* = 2 to 12, and chose the number of stages (*k*) with the maximum of the average silhouette.

### Selection of stage-specific seRNAs

We decomposed the seRNA expression profiles using NMF and obtained the seRNA vs. stage matrix that contained a column for each stage and a row for each seRNA (as mentioned above). We converted this seRNA vs. stage matrix into a difference matrix by scaling the values of each stage to unity mean and subtracting the maximum value for other stages. The seRNAs with a difference greater than two times the standard deviation of the differences in a given stage were defined as stage-specific seRNAs.

### Identification of seRNA-associated genes via seRNA-mRNA coexpression network

To identify the seRNA-associated genes, we constructed a seRNA-mRNA coexpression network. First, the absolute values of the Pearson’s correlation coefficient (PCC) among seRNAs and mRNAs were calculated. Next, for each pair, seRNA A and mRNA B, the mutual rank (MR) index was calculated as the geometric average of the PCC rank from A to B and that from B to A [21]. mRNAs with mutual ranks to a seRNAs of ≤5 were determined to be the associated mRNAs of the given seRNA .

### Transcription factor over-representation analysis

Transcriptional factor binding sites (TFBSs) were obtained via the Table Browser of the UCSC Genome Browser (http://genome.ucsc.edu/) from the “Txn Factor ChIP” track (table name: wgEncodeRegTfbsClusteredV3). This dataset was generated by ENCODE Analysis Working Group which uniformly processed the ENCODE ChIP-seq data for 161 transcription factors in 91 cell types and combined the identified peaks into clusters to produce a summary display.

An eRNA was defined as a target of a specific TF if the binding site of the given TF fell within 500 bp upstream or downstream of the given eRNA locus. Similarly, a gene was considered to be a target gene of a specific TF if the binding site of the given TF fell within the promoter of the given gene. Promoters were defined as the upstream and downstream 500 bp of a transcription start site (TSS).

To assess whether the binding sites of a specific TF were over-represented in a set of genomic regions of interest, such as seRNA loci or the promoters of seRNA-associated genes, a one-sided Fisher's exact test was performed using a 2 × 2 contingency table. This test and the table include the following numbers: *n*, *N − n*, *r*, *R − r*, where *n* denotes the number of target seRNAs or genes of the given TF, *N* denotes the number of seRNAs or genes of interest, *R* is equal to *N,* and *r* denotes the mean number of randomly selected *R* seRNAs or genes which are also the target of the given TF after 1000 rounds. TFs with a *P*-value of < 0.05 in these tests were defined as enriched TFs.

### Gene ontology over-representation analysis

Gene ontology (GO) over-representation analysis was applied to each group of seRNA-associated genes using the Bioconductor package topGO [25], with the ‘classic’ algorithms and the Benjamini–Hochberg procedure for multiple test correction. We only focused on the GO terms of biological process ontology with FDR < 0.05. The statistically over-represented GO terms were visualized by REVIGO with ‘SimRel’ semantic similarity measurement [42].

### Function-association network of seRNA-associated genes

The function-association network of the seRNA-associated genes was constructed using the STRING database (version 10.5) [43]. Each query consists of a group of associated genes and the significantly bound TFs. The required interaction score for connecting nodes was set to “low confidence (0.150)”.

### CTCF ChIP-seq datasets

The processed ChIP-seq (BigWig format) of CTCF in human embryonic stem cells (ESC) and ESC-derived cells were download from ChIP-Atlas [44] with accession number SRX378281, SRX378282, SRX378283, SRX378284, and SRX378285. The processed datasets were visualized by the Integrative Genomic Viewer (IGV) [45].

## Supplementary information

**Additional file 1: Figure S1.** Average silhouette scores with various number of stages for NMF decomposition of the unstitched (A) and stitched seRNA profiles (B). **Figure S2.** Location distribution of associated genes for early stage-specific seRNAs. Bar plot showing the number of associated genes and scatter plot showing the distance between associated genes and their seRNAs. The distance is defined as the absolute difference between two locus midpoints. The number of associated genes that located on the same chromosome as their seRNA is indicated above the scatter plot. **Figure S3.** The regulator binding matrix of early-stage-specific seRNA-associated genes. Heatmap visualizing the results of TF over-representation analysis on seRNA-associated genes. Red borders indicate that the TF also binds to the super-enhancer. The color denotes −log10 of the *P*-value obtained by the Fisher’s exact test. (* *P* < 0.05). **Figure S4.** Normalized ChIP-seq tracks for CTCF demonstrate a stronger CTCF-binding at the seRNA (chr17:72764600–72,764,690) in the human embryonic stem cells (hESC), comparing to hESC-derived cells.

**Additional file 2.** Lists of identified stage-specific seRNAs.

**Additional file 3.** GO terms of early stage-specific seRNA-associated genes (All pooled).

**Additional file 4.** GO terms of late stage-specific seRNA-associated genes (All pooled).

**Additional file 5.** GO terms of individual late stage-specific seRNA-associated genes, related to Fig. 5.

**Additional file 6.** GO terms of individual early stage-specific seRNA-associated genes, related to Additional file 1: Figure S2.

## Data Availability

The CAGE-seq dataset is available via the FAMTOM5 website (http://fantom.gsc.riken.jp/5/tet/data/hg19.cage_peak_phase1and2combined_tpm_ann_decoded.osc.txt.gz). The scripts used for analyses have been deposited at GitHub and are available at https://github.com/haochunchang/seRNA-ESC.
