# Supplementary material for: Investigating the role of super-enhancer RNAs underlying embryonic stem cell differentiation
Source: BMC Genomics. 2019 Dec 30;20(Suppl 10):896. doi: 10.1186/s12864-019-6293-x (PMC6936076; doi:10.1186/s12864-019-6293-x)
Supplement: Supplementary file 1 — Additional file 1: Figure S1. Average silhouette scores with various number of stages for NMF decomposition of the unstitched (A) and stitched seRNA profiles (B). Figure S2. Location distribution of associated genes for early stage-specific seRNAs. Bar plot showing the number of associated genes and scatter plot showing the distance between associated genes and their seRNAs. The distance is defined as the absolute difference between two locus midpoints. The number of associated genes that located on the same chromosome as their seRNA is indicated above the scatter plot. Figure S3. The regulator binding matrix of early-stage-specific seRNA-associated genes. Heatmap visualizing the results of TF over-representation analysis on seRNA-associated genes. Red borders indicate that the TF also binds to the super-enhancer. The color denotes −log10 of the P-value obtained by the Fisher’s exact test. (* P < 0.05). Figure S4. Normalized ChIP-seq tracks for CTCF demonstrate a stronger CTCF-binding at the seRNA (chr17:72764600–72,764,690) in the human embryonic stem cells (hESC), comparing to hESC-derived cells. [file 12864_2019_6293_MOESM1_ESM.pdf]

Additional file 1 – Supplementary figures

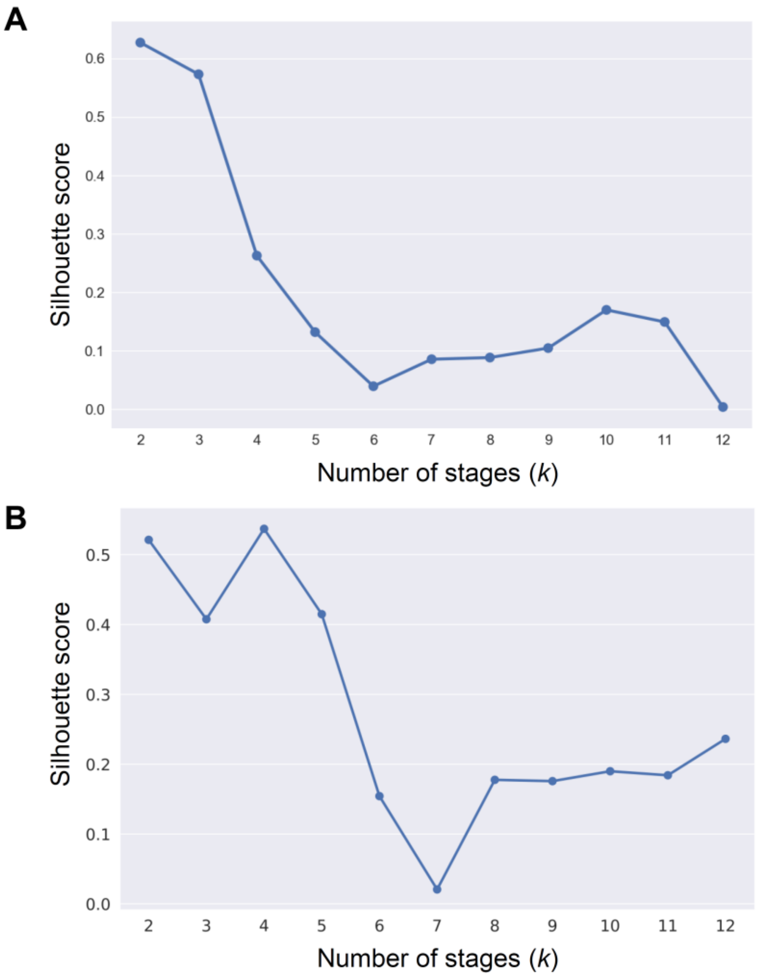

Figure S1 – Average silhouette scores with various number of stages for NMF decomposition of the unstitched (A) and stitched seRNA profiles (B)

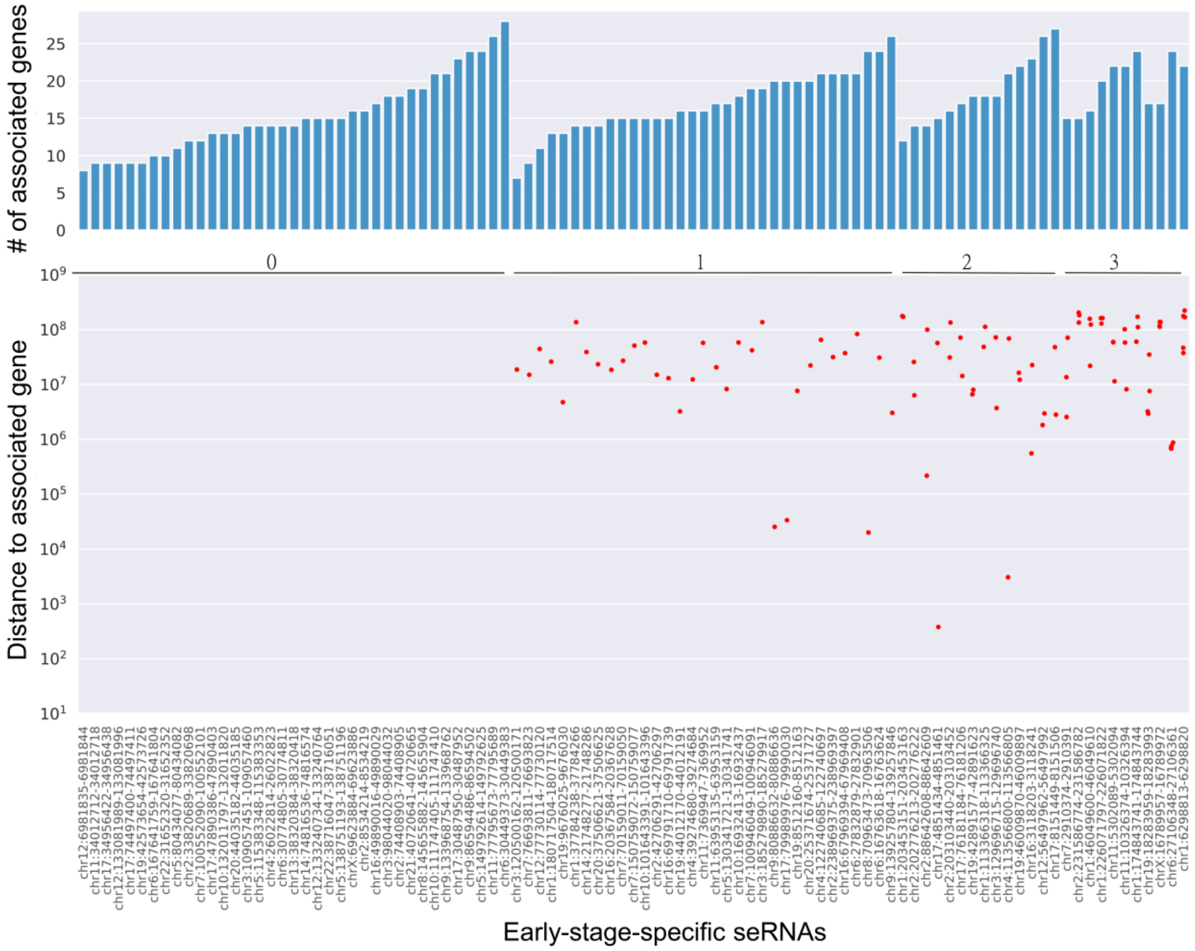

Figure S2 - Location distribution of associated genes for early stage-specific seRNAs

Bar plot showing the number of associated genes and scatter plot showing the distance between associated genes and their seRNAs. The distance is defined as the absolute difference between two locus midpoints. The number of associated genes that located on the same chromosome as their seRNA is indicated above the box plots.

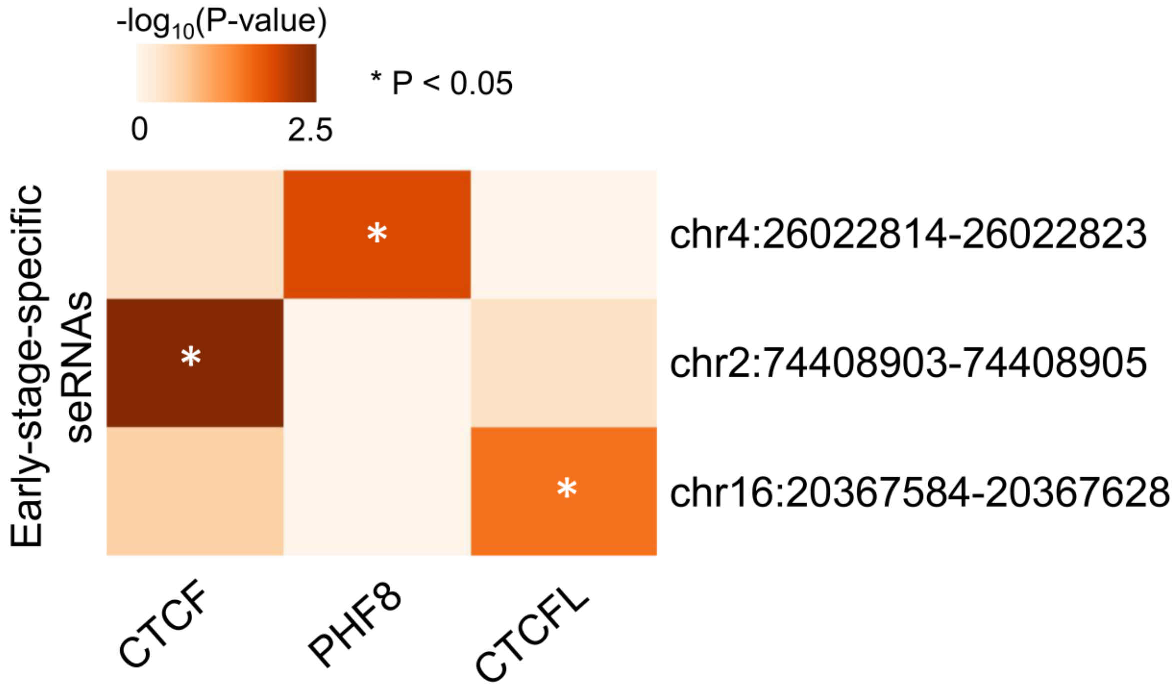

Figure S3 - The regulator binding matrix of early-stage-specific seRNA-associated genes. Heatmap visualizing the results of TF over-representation analysis on seRNA-associated genes. Red borders indicate that the TF also binds to the super-enhancer. The color denotes −log10 of the P-value obtained by the Fisher's exact test. (* $P < 0.05$).

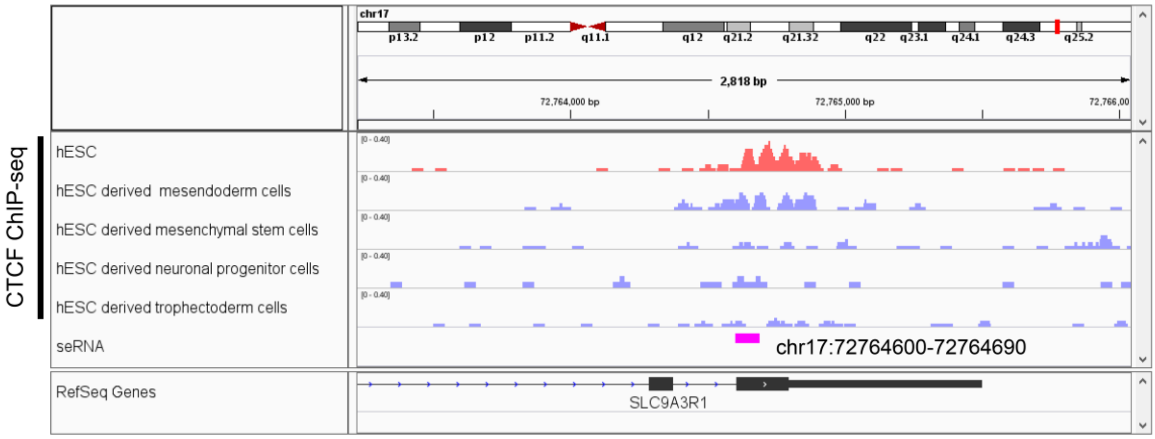

Figure S4 - Normalized ChIP-seq tracks for CTCF demonstrate a stronger CTCF-binding at the seRNA (chr17:72764600-72764690) in the human embryonic stem cells (hESC), comparing to hESC-derived cells.
